# A Case of Brain Metastases from Breast Cancer Treated with Whole-Brain Radiotherapy and Eribulin Mesylate

**DOI:** 10.1155/2012/537183

**Published:** 2012-08-16

**Authors:** Carsten Nieder, Gro Aandahl, Astrid Dalhaug

**Affiliations:** ^1^Department of Oncology and Palliative Medicine, Nordland Hospital, P.O. Box 1480, 8092 Bodø, Norway; ^2^Institute of Clinical Medicine, Faculty of Health Sciences, University of Tromsø, N-9037 Tromsø, Norway

## Abstract

Patients with triple receptor-negative breast cancer often develop aggressive metastatic disease, which also might involve the brain. In many cases, systemic and local treatment is needed. It is important to consider the toxicity of chemo- and radiotherapy, especially when newly approved drugs become available. Randomised studies leading to drug approval often exclude patients with newly diagnosed brain metastases. Here we report our initial experience with eribulin mesylate and whole-brain radiotherapy (WBRT) in a heavily pretreated patient with multiple brain, lung, and bone metastases from triple receptor-negative breast cancer. Eribulin mesylate was given after 4 previous lines for metastatic disease. Two weeks after the initial dose, that is, during the first cycle, the patient was diagnosed with 5 brain metastases with a maximum size of approximately 4.5 cm. She continued chemotherapy and received concomitant WBRT with 10 fractions of 3 Gy. After 3 cycles of eribulin mesylate, treatment was discontinued because of newly diagnosed liver metastases and progression in the lungs. No unexpected acute toxicity was observed. The only relevant adverse reactions were haematological events after the third cycle (haemoglobin 9.5 g/dL, leukocytes 3.1 × 10^9^/L). The patient died from respiratory failure 18.5 months from diagnosis of metastatic disease, and 2.7 months from diagnosis of brain metastases. To the best of our knowledge, this is the first report on combined WBRT and eribulin mesylate.

## 1. Introduction

Development of brain metastases is one of the major challenges in patients with stage III and IV breast cancer. In a recent study, brain metastases-free survival differed significantly between breast cancer subtypes and was shortest in patients with triple receptor-negative cancer [1]. A large study in patients with triple receptor-negative breast cancer showed that 9.6% of those with initial stages III disease developed brain metastases as first site of recurrence within 5 years [2]. The total incidence is even higher because most recurrences are found in other distant sites before the disease spreads to the brain. Systemic therapy is needed to control widespread disease outside the brain, while radiotherapy and/or surgical resection typically are used to treat brain metastases [3–5]. When new chemotherapy options become available, one important question regarding the sequence of treatments has to be addressed: is it safe to combine radiotherapy and systemic chemotherapy? Here, we report our initial experience with eribulin mesylate in a patient with extracranial and multiple brain metastases treated with whole-brain radiotherapy (WBRT). 

## 2. Case Report 

In July 2009, a 57-year-old Caucasian female was treated for poorly differentiated triple receptor-negative invasive ductal carcinoma in her left breast. She had detected a lump in her breast approximately one month before surgery (lumpectomy and sentinel node procedure). Her previous medical history and family history was unremarkable. Pathological TNM stage was T2 (25 mm diameter) N0 (four lymph nodes examined). Proliferation index was high (Ki-67 55%). According to national guidelines, she received adjuvant chemotherapy (epirubicin, cyclophosphamide, 5-fluorouracil and FEC-60 regimen) and radiotherapy (breast tangents only, 25 fractions of 2 Gy). 

**Figure 1:**
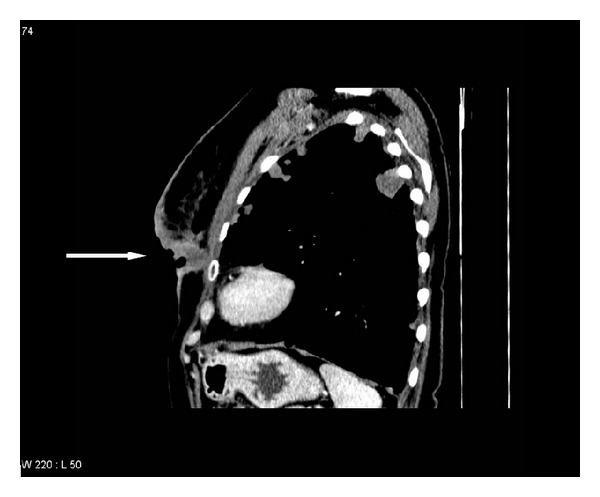
Sagittal computed tomography scan of the chest showing local relapse in the lower inner quadrant of the breast after breast conserving surgery and adjuvant treatment.

**Figure 2:**
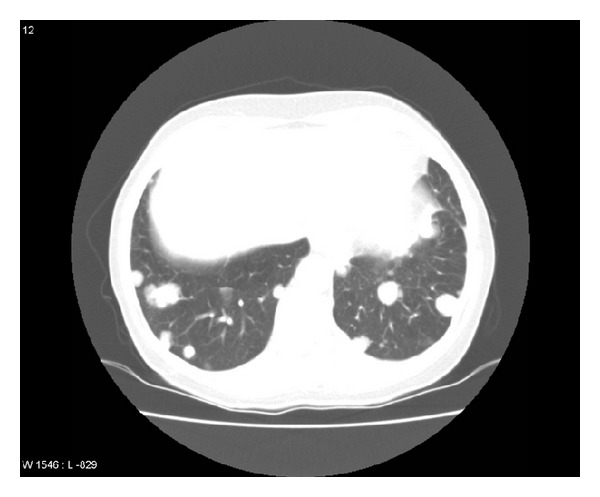
Computed tomography scan of the chest showing multiple bilateral lung metastases.

In August 2010, she was diagnosed with multiple bilateral lung metastases and bone metastases. Blood chemistry including alkaline phosphatase and lactate dehydrogenase was unremarkable. Treatment with zoledronic acid 4 mg every 4 weeks and palliative chemotherapy with Taxotere was initiated (100 mg/m^2^ every 3 weeks). After 5 cycles, progression of lung metastases plus local relapse in the left breast with small satellite nodules on the skin was detected. Therefore, in December 2010, chemotherapy was switched to oral vinorelbine 60 mg/m^2^ and after 3 weeks the dose was increased to 80 mg/m^2^. After 2 months on this regimen, progression in the lungs and breast was obvious. In February 2011, chemotherapy was switched to oral capecitabine 2500 mg/m^2^. Due to further progression by the end of March, a new regimen was started (carboplatin AUC 4 plus gemcitabine 800 mg/m^2^ day 1 and 8 every 3 weeks, zoledronic acid was still continued) and the patient was also referred for reirradiation to the left breast because of ulcerating local relapse in the lower inner quadrant (Figure 1). After 2 cycles, while waiting for radiotherapy, the first episode of high grade toxicity during the whole course of treatment developed (haemoglobin 8.2 g/dL and thrombocytes 75 ×10^9^/L). The patient received red blood cell transfusion and started palliative reirradiation afterwards. Ten fractions of 3 Gy were given, immediately followed by the third cycle of chemotherapy. After 8 cycles, blood transfusion was necessary again (haemoglobin 9.3 g/dL). At that time, disease in the lungs and bones was stable and partial response in the breast was obtained. After 9 cycles, in November 2011, the patient was admitted to the hospital because of abdominal pain. She was diagnosed with mechanic ileus and required surgery (removal of bowel adhesions, unrelated to breast cancer, and quick recovery). However, progression of lung metastases was detected (Figure 2). 

**Figure 3:**
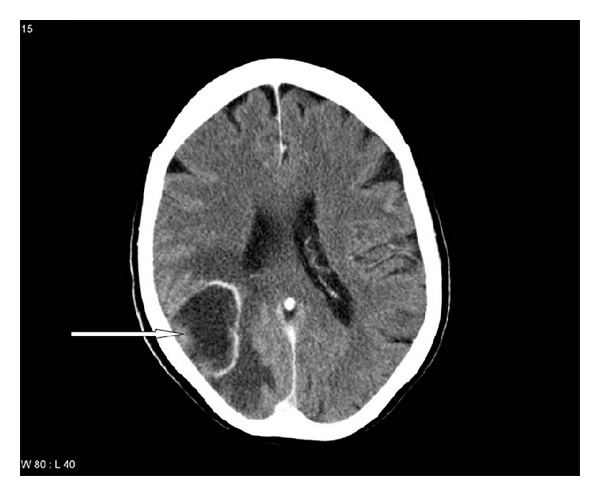
Contrast-enhanced computed tomography of the brain showing a 4.5 cm large, partially necrotic brain metastasis with oedema (overall 5 metastases were present).

After 4 different lines with chemotherapy for metastatic disease with stable disease as best outcome, eribulin mesylate was chosen (1.4 mg/m^2^ day 1 and 8 every 3 weeks). Two weeks after the initial dose, that is, during the first cycle, the patient developed increasing headache and gait disturbance. Computed tomography showed 5 brain metastases with a maximum size of approximately 4.5 cm (Figure 3). No screening for brain metastases had been performed during the previous year. The patient had a Karnofsky performance status (KPS) of 70. Given the size of the lesions, radiosurgery was not an option. WBRT was administered (10 fractions of 3 Gy), together with decreasing doses of corticosteroids. Acute side effects were limited to mild skin toxicity and fatigue. In parallel, the patient received chemotherapy (second cycle), which also was continued after WBRT. Evaluation after 3 cycles unfortunately showed progression of lung metastases and, for the first time, liver metastases were found. Haematological toxicity after 3 cycles was limited (haemoglobin 9.5 g/dL, no transfusion given and leukocytes 3.1 × 10^9^/L). No other high grade toxicity was detected. The patient discontinued treatment because of disease progression in February 2012 and died from respiratory failure in the hospital one month later. Survival was 31 months from initial breast surgery, 18.5 months from diagnosis of metastatic disease, and 2.7 months from diagnosis of brain metastases. 

## 3. Discussion

Eribulin mesylate is a recently approved new therapeutic option for patients with metastatic breast cancer [6]. In the pivotal phase III study, patients with brain metastases were excluded unless these metastases were treated and stable [7]. The number of patients with previously treated brain metastases in the trial was not reported. Nineteen percent of patients had triple receptor-negative disease. Median duration of treatment was 3.9 months. Median progression-free survival was 3.7 months. The objective response rate was 12% and 41% of patients had progressive disease as their best overall tumour response. Common grade 3 or 4 adverse events included neutropenia, leukopenia and peripheral, neuropathy. Previous phase II studies also excluded patients with active symptomatic brain metastases or progression of known brain metastases [8, 9]. Again, the number of patients with brain metastases included in these trials was not reported. It is therefore important to evaluate the tolerability of eribulin mesylate in patients who undergo treatment for active brain metastases. In the case reported here, both drug treatment and WBRT was tolerated without high grade toxicity. Haematological toxicity was acceptable. However, after 3 cycles treatment was discontinued due to lack of efficacy. Therefore, long-term data can not be provided. Given the aggressive biology of metastatic triple receptor-negative breast cancer [10] and heavy pretreatment of our patient, lack of response was not unexpected. Our patient derived most benefit in terms of disease stabilisation from carboplatin plus gemcitabine chemotherapy. Survival after diagnosis of brain metastases was limited to 2.7 months. This figure is in agreement with our previous experience in patients with adverse prognostic features such as progressive systemic disease, short interval between primary diagnosis and brain metastases, and KPS < 80 [11, 12]. Due to size and number of brain metastases neither surgical resection nor radiosurgery could be offered. In patients with less extensive brain involvement, these options should be considered because they provide superior local control. It might have been unfortunate that our patient did not undergo screening for brain metastases earlier during the course of disease. However, the final outcome with death from respiratory failure in the presence of extensive bilateral lung metastases probably would have been the same. As illustrated here and in recent studies [4, 13], it is important to achieve optimal extracranial control and to provide efficacious systemic treatment even after the diagnosis of brain metastases.
